# Small molecule inhibitors provide insights into the relevance of LAT1 and LAT2 in materno‐foetal amino acid transport

**DOI:** 10.1111/jcmm.15840

**Published:** 2020-10-01

**Authors:** Jonas Zaugg, Xiao Huang, Fabian Ziegler, Matthias Rubin, Julien Graff, Jennifer Müller, Ruedi Moser‐Hässig, Theresa Powell, Jürg Gertsch, Karl‐Heinz Altmann, Christiane Albrecht

**Affiliations:** ^1^ Institute of Biochemistry and Molecular Medicine Faculty of Medicine University of Bern Bern Switzerland; ^2^ Swiss National Centre of Competence in Research (NCCR) TransCure University of Bern Bern Switzerland; ^3^ Institute of Pharmaceutical Sciences Department of Chemistry and Applied Biosciences ETH Zurich Zurich Switzerland; ^4^ Division of Gynecology and Obstetrics Lindenhofgruppe Bern Switzerland; ^5^ Department of Pediatrics, Neonatology Section University of Colorado Denver CO USA

**Keywords:** BeWo, LAT1 (SLC7A5), LAT2 (SLC7A8), leucine uptake, monolayer, placenta, transplacental amino acid transport, Transwell, trophoblast, trophoblast differentiation

## Abstract

The placenta supplies the foetus with critical nutrients such as essential amino acids (AA, eg leucine) for development and growth. It also represents a cellular barrier which is formed by a polarized, differentiated syncytiotrophoblast (STB) monolayer. Active Na^+^‐independent leucine transport across the placenta is mainly attributed to the System L transporters LAT1/SLC7A5 and LAT2/SLC7A8. This study explored the influence of trophoblast differentiation on the activity of LAT1/LAT2 and the relevance of LAT1/LAT2 in leucine uptake and transfer in trophoblasts by applying specific small molecule inhibitors (JPH203/JG336/JX009). L‐leucine uptake (total dose = 167 μmol/L) was sensitive to LAT1‐specific inhibition by JPH203 (EC_50_ = 2.55 µmol/L). The inhibition efficiency of JPH203 was increased by an additional methoxy group in the JPH203‐derivate JG336 (EC_50_ = 1.99 µmol/L). Interestingly, JX009 showed efficient System L inhibition (EC_50_ = 2.35 µmol/L) and was the most potent inhibitor of leucine uptake in trophoblasts. The application of JPH203 and JX009 in Transwell^®^‐based leucine transfer revealed LAT1 as the major accumulative transporter at the apical membrane, but other System L transporters such as LAT2 as rate‐limiting for leucine efflux across the basal membrane. Therefore, differential specificity of the applied inhibitors allowed for estimation of the contribution of LAT1 and LAT2 in materno‐foetal AA transfer and their potential impact in pregnancy diseases associated with impaired foetal growth.

## 1 INTRODUCTION

**FIGURE 1:**
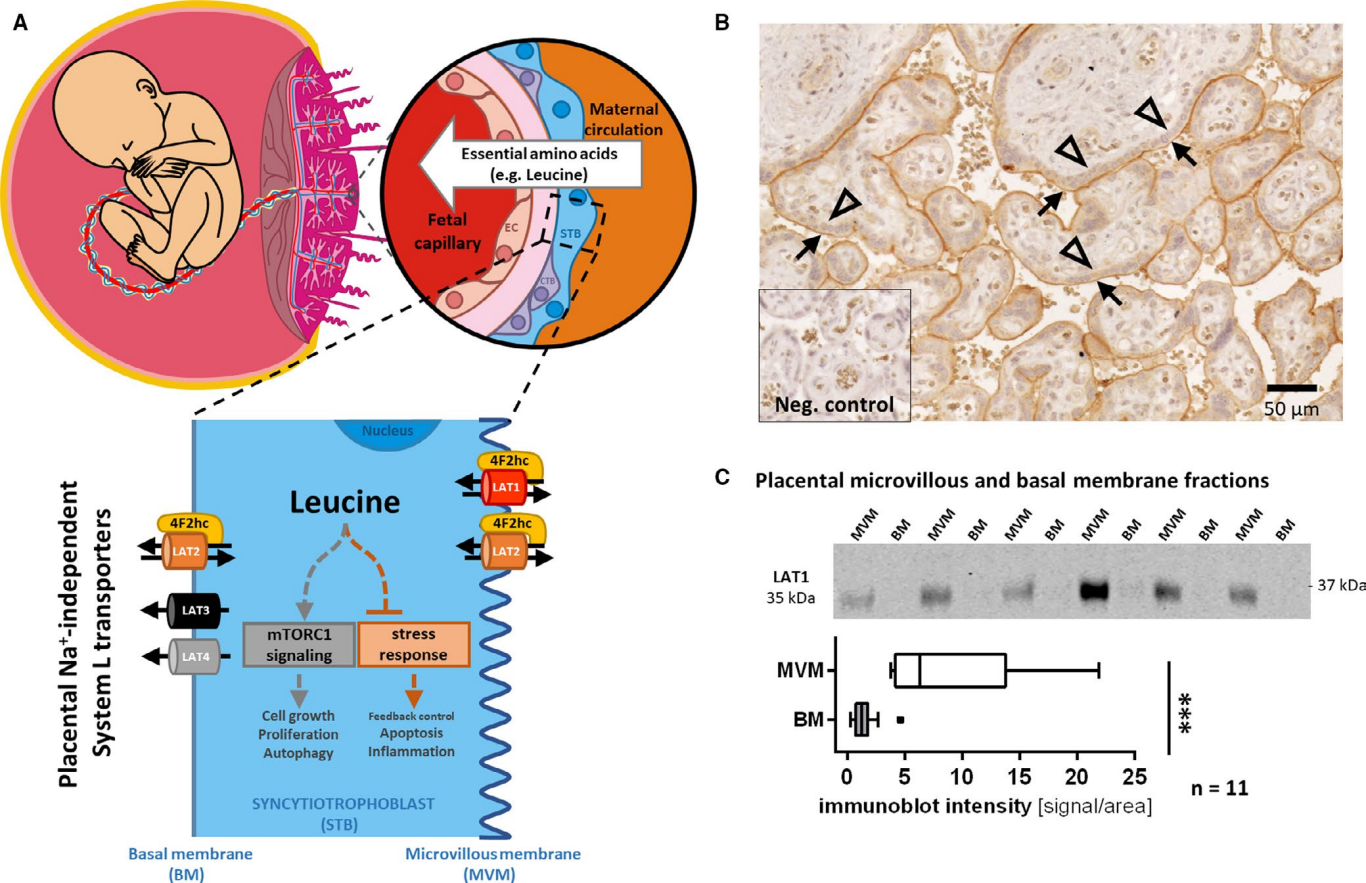
Schematic representation of Na^+^‐independent leucine transfer across the placental barrier with asymmetric LAT1 expression. Scheme of the placental villous structures as interface between the foetal capillaries and the maternal circulation (A, upper left panel) and the human placental barrier (A, upper right panel) involving three different cell types: endothelial cells lining the foetal capillaries (EC), varying intercellular space (pink), single‐nucleated cytotrophoblasts (CTB) and syncytiotrophoblasts (STB) forming a multi‐nucleated monolayer, which is in direct contact with maternal blood and mainly responsible for materno‐foetal nutrient transport. In (A) lower panel, Na^+^‐independent System L exchanger and System L‐like facilitators known to be expressed in the human placenta are shown. They are present either at the apical microvillous membrane (MVM) or at the basal membrane (BM) of STB. LAT1 (SLC7A5), expressed at the MVM, and LAT2 (SLC7A8), present at both the MVM and BM, are SLC7 family members and colocalize with their heavy chain partner 4F2hc (SLC3A2, CD98). LAT3 (SLC43A1) and LAT4 (SLC43A2) were described as System L‐like facilitators and are most likely expressed at the BM. Increased intracellular leucine concentrations stimulate mTORC1‐mediated cell proliferation and survival, and repress the ATF4‐mediated amino acid balance sensing system, which reduces global translation and increases biosynthesis. (B) Representative picture showing apical LAT1 localization in immunohistochemistry of human term placenta. Arrows indicate the apical MVM, that is, maternal blood orientated side; arrowheads depict the BM of the STB. There was no signal in negative control. (C) upper panel, representative immunoblot of 6 purified membrane protein samples isolated from term placental tissues. (C) lower panel, LAT1 was highly expressed in MVM, but negligible in BM protein fractions isolated from a total of 11 human term placentae. The data is normalized to the respective tissue homogenate before membrane separation and shown as boxplots with Tukey whiskers (Mann‐Whitney test, α = 0.05; ***P < 0.0001)

Adequate amino acid (AA) supply is vital especially for highly proliferative tissues like the placenta. Other tissues which are critical for nutrient absorption and transport, such as the intestine and liver, mainly use the efficient sodium (Na^+^)‐dependent uptake maintained by System A transporters,^1^ such as the SNAT family.^2^ The heteromeric SLC7 AA transporters, a subgroup of the System L‐exchanger family, are driven by System A‐dependent AA gradients. Both AA transporter families are highly expressed in polarized epithelial tissues such as blood‐brain and placental barriers.^3^, ^4^ There are two important cell layers in the placental villi coordinating the nutrient transfer across the placental barrier, namely syncytiotrophoblasts (STB) and foetal capillary endothelial cells. Endothelial cells lining the foetal vessels allow relatively unrestricted paracellular diffusion of small molecules like glucose and AA through endothelial junctions.^5^ In contrast, STB represent the limiting barrier for AA due to the formation of an epithelial syncytium composed of two polarized monolayers, the microvillous plasma membrane (MVM) facing the maternal blood supply and the basal membrane (BM) directed towards the foetal capillary (Figure 1A).

The Na^+^‐independent System L transporters expressed in the human placenta are heterodimeric exchangers consisting of the light chain L‐type AA transporter LAT1 (SLC7A5) or LAT2 (SLC7A8) covalently attached to the heavy chain 4F2hc (SLC3A2). Moreover, the SLC43 family members LAT3 (SLC43A1) and LAT4 (SLC43A2), known to be involved in facilitated AA diffusion,^6^, ^7^ are expressed at the BM (Figure 1A). Both LAT1 and LAT2 are predominantly localized to the MVM of human term placenta, LAT2 as well as LAT3 and LAT4 are also present at the BM and in endothelial cells lining the foetal capillaries.^7^, ^8^ In the last decade, increasing evidence suggests a tight link between the reduced activity of placental System L transporters and intrauterine growth restriction (IUGR),^9^, ^10^ and their up‐regulation in placentae of large for gestational age (LGA) infants.^11^ Such altered foetal development has a fundamental impact on lifelong health and wellbeing, and may contribute by foetal programming to an increased prevalence for cardiovascular disease and diabetes/adiposity later in life.^12^, ^13^, ^14^, ^15^ Notably, it has been reported that LAT1 or its associated glycoprotein 4F2hc is involved in placenta decidualization and fusogenic trophoblast differentiation.^16^ This could imply that the diminished leucine uptake found in knock‐down cell models^17^ results rather from failure in trophoblast differentiation than from reduced SLC7 transport activity. Hence, small molecules which induce only short‐term inhibition of transporter activity are a valuable experimental tool to study placental transfer mechanisms as they will not affect trophoblast differentiation and associated processes like trophoblast fusion. Therefore, studying placental AA transfer by using specific small molecule inhibitors of AA transporters instead of silencing or knock‐out could help to reveal the relevance of LAT1 or LAT2 in materno‐foetal leucine transfer without affecting cell differentiation.

Since LAT1 was found to be selectively expressed and up‐regulated in various rapidly proliferative cancer types^18^, ^19^, ^20^ and has a putative role in drug delivery across the blood‐brain barrier,^21^ efforts have been made to pharmaceutically target this transporter using substrate‐mimicking or virtual screening approaches.^22^, ^23^, ^24^, ^25^, ^26^ The substrate‐mimicking tyrosine analog JPH203 (also known as KYT‐0353) was tested in several in vitro and in vivo cancer cell proliferation experiments and described as potent LAT1‐specific inhibitor.^27^, ^28^, ^29^, ^30^, ^31^ To delineate the contribution of LAT1 from LAT2 in materno‐foetal leucine transport, we synthesized the LAT1‐specific inhibitor JPH203, the structurally closely related inhibitor (JG336), as well as a third small molecule inhibitor (JX009) with comparable leucine uptake inhibition efficiency but lower LAT1‐specificity (structures see Figure 4).

In this study, we assessed the contribution of LAT1‐mediated Na^+^‐independent leucine uptake into trophoblasts and investigated the transfer of this essential AA across the placental barrier by transient inhibition. To achieve these goals, we (a) tested primary human trophoblast and BeWo (clone b30) cell models for LAT1, LAT2 and 4F2hc expression and leucine uptake capacity under Na^+^‐free conditions, (b) investigated whether the differentiation status of primary trophoblasts and BeWo cells has an impact on leucine uptake and (c) assessed the contribution of LAT1 for the uptake and transfer of leucine by application of small molecule inhibitors.

## 2 MATERIALS AND METHODS

All chemicals and reagents were purchased from Sigma‐Aldrich in Switzerland unless otherwise stated.

### 2.1 Isolation of primary human trophoblast cells

Placentae from normal healthy pregnancies were collected after elective Caesarean section at the Division of Gynecology and Obstetrics, Lindenhofgruppe, Bern, Switzerland. Details on the study subjects are found in Table 1. The study was conducted in accordance with the Declaration of Helsinki, and the protocol was approved by the Ethics Committee of the Canton of Bern (Basec Nr2016‐00250). The collected tissue was used to isolate primary cytotrophoblasts as previously described.^32^ Isolated cells were cultured on Cell‐BIND plates in Dulbecco's modified Eagle's medium containing 4.5 g/L glucose (DMEM‐highGlucose; Gibco, Paisley, UK) and characterized by analysing the expression of cytokeratin‐7 and vimentin as previously described.^33^ Primary trophoblasts were evaluated for leucine uptake at the cytotrophoblast (CTB) and syncytiotrophoblast (STB) stage (ie after 12 and 48 hours of culture, respectively) when spontaneous differentiation and fusion has occurred.

**TABLE 1:** Anthropometric characteristics of healthy patients donating placental tissue and their offspring

	Characteristics	Healthy controls
Mother	Number of individuals	11
	Maternal age (y)	33.9 ± 3.48
	Parity	1.8 ± 0.60
	Gestational age at partum	39 3/7 ± 6/7
Newborn	Weight of placenta (g)	569.3 ± 86.1
	Weight of baby (g)	3367.3 ± 284.0
	Sex of baby	3♂/6♀

### 2.2 BeWo cell culture

BeWo cells (clone b30) were cultured in DMEM containing 1.0 g/L glucose (DMEM‐lowGlucose; Gibco). BeWo cells require stimulation by forskolin to induce STB formation.^34^ Unstimulated BeWo cells representing the CTB stage were studied after 24 hours of culture; differentiated, syncytialized BeWo cells were analysed after stimulation with 100 μmol/L forskolin for 48 hours when markers for syncytialization are significantly increased.^32^ For leucine transfer experiments, BeWo cells were seeded at a density of 100 000 cells/cm^2^ onto permeable membranes (0.4 μm pore size) mounted in 12‐well format from the Transwell^®^ system (Corning Inc., Corning, NY, USA). Cells were cultured in DMEM‐lowGlucose at 37°C with 5% CO_2_ atmosphere for 7‐14 days.

### 2.3 Placental membrane protein isolation

Placental tissues from healthy pregnancies were used to simultaneously isolate microvillous membranes (MVM) and basal membranes (BM) by Mg^2+^ precipitation based on a published method^35^ and described in the Supporting Information. Expression levels in MVM and BM were normalized to the total membrane isolation (TMI) fraction collected prior to Mg^2+^ precipitation for MVM/BM separation. Further details regarding the enrichment and characterization of the MVM/BM fractions are found in Supporting Information and Figure S1.

### 2.4 LAT1 localization by immunohistochemistry and immunoblotting

LAT1 protein was localized in two experimental approaches. LAT1 expression in MVM and BM fractions was determined by immunoblotting using rabbit polyclonal anti‐LAT1 antibody (Anti‐human LAT1, KE026/TG170215 Transgenic Inc., Kobe, Japan; 1:1000). For the reference signal (loading control) a mouse anti‐beta‐actin antibody (A2228, Sigma Aldrich, Buchs SG, Switzerland) was used. Details regarding the immunoblotting procedures are described in Supporting Information. Densitometrical analysis of immunoblots was performed using the LI‐COR OdysseyW Imaging System. LAT1 expression in placental tissue was visualized by immunohistochemistry using the same LAT1‐specific antibody (10 µg/mL). The preparation of cryosections and the staining procedure including visualization are described in Supporting Information.

### 2.5 Expression changes of LAT1, LAT2 and 4F2hc during trophoblast differentiation

To compare changes in expression of LAT1, LAT2 and 4F2hc during cell differentiation, mRNA and protein levels were determined by RT‐qPCR and immunoblotting, respectively. RNA isolation, first‐strand cDNA synthesis and RT‐qPCR analysis were performed as previously described.^36^ Expression results were normalized to the mean of the reference genes YWHAZ, GAPDH and β‐actin. Primer nucleotide sequences are listed in Table S1. For protein quantification 40 µg cell lysates were loaded on 10% acrylamide gels and separated by SDS–PAGE. The immobilized bands were semi‐dry transferred to nitrocellulose membranes (GE Healthcare, Glattbrugg, Switzerland). Blots were blocked with 5% w/v non‐fat milk in Tris Buffered Saline with 0.1% Tween‐20 (TBST). Proteins were detected by antibodies against LAT1 (see above), LAT2 (Anti‐SLC7A8, AV43930, Sigma‐Aldrich) and 4F2hc (CD98 (E‐5), sc‐376815, Santa Cruz, Biotechnology Inc., Heidelberg, Germany). Protein content was measured using a commercial Pierce™ BCA Protein Assay Kit.

### 2.6 Leucine uptake assay

The leucine uptake protocol was based on a recently published method^30^ and adapted to physiological leucine concentrations. In brief, BeWo cells were seeded at a density of 60 000 cells/well into white‐walled 96‐well plates (Corning). The isolated primary trophoblasts were seeded at a density of 100 000 cells/well on 96‐well plates which were coated with Matrigel (BD, New Jersey, USA) according to the suppliers' instructions. Primary trophoblasts and BeWo cells were cultured until they reached their assigned differentiation stage and then washed 3‐times with pre‐warmed Na^+^‐free Hank's buffer (125 mmol/L choline chloride, 25 mmol/L HEPES, 4.8 mmol/L KCl, 1.2 mmol/L MgSO_4_, 1.2 mmol/L KH_2_PO_4_, 1.3 mmol/L CaCl_2_, 5.6 mmol/L glucose, adjusted pH to 7.4). After equilibration in the same buffer (37°C, 7 minutes), cells were incubated for 3 minutes with pre‐warmed Na^+^‐free Hank's buffer containing 167 μmol/L L‐leucine and 20 nmol/L radioactive L‐[3,4,5‐^3^H(N)]‐leucine (PerkinElmer, Waltham, MA, USA). Leucine uptake was stopped by three washing steps with ice‐cold Na^+^‐free Hank's buffer. The use of Na^+^‐free buffer prevents leucine uptake by other Na^+^‐dependent transporters such as System A‐family members. Cells were lysed by intense shaking for 1.5 hours in MicroScint™‐20 scintillation liquid (PerkinElmer). ^3^[H]‐leucine was quantified using TopCount^®^ NXT™ Scintillation and Luminescence Counter (PerkinElmer).

### 2.7 Specific inhibition of LAT1/2 mediated placental leucine uptake

JPH203, JG336 and JX009 were synthesized following the routes described in the European Patent Application EP 2‐959‐918‐A1, 2014. Compounds were obtained as hydrochlorides, dissolved in dimethyl sulfoxide (DMSO, Merck, Darmstadt, Germany) and applied in the concentration range from 0.001 to 31.6 μmol/L. JPH203 has been characterized as highly LAT1‐specific; JX009 is described as LAT1 and LAT2‐specific inhibitor.^37^ JG336 is a derivate of JPH203 with an additional methoxy group at the peripheral phenyl moiety residue. Compound structures are depicted in Figure 4. The ^1^H‐NNMR and ^13^C‐NMR spectra data of the compounds were in agreement with the expected structures and are included in the Supporting Information.

### 2.8 Leucine transfer across a polarized trophoblast monolayer

Before starting the transfer experiment, the formation of a tight trophoblast monolayer in BeWo cells was monitored by measuring transepithelial electrical resistance (TEER, [Ω*cm^2^]) and passive diffusion as reported previously.^38^ TEER and cellular capacitance (C_cl_, [µF/cm^2^]) were measured every 30 minutes and analysed using the cellZscope system (nanoAnalytics, Münster, Germany) according to the manufacturer's instructions. The apparent permeability coefficient (Papp, [cm/s]) was calculated by measuring the rates of passive transfer of the paracellular pathway marker Lucifer yellow (LY) as previously described.^39^ BeWo monolayers with verified tightness were selected after 7 days of culturing on Transwell^®^ membranes and randomly assigned to leucine transfer time courses and inhibition experiments. The inserts with cultured BeWo cells and an insert without cells (no cell control) were placed in 12‐well plates and washed 3‐times with pre‐warmed Na^+^‐free Hank's buffer. Prior to starting the time course, the cells were equilibrated in Hank's buffer for 30 minutes at 37°C. Leucine transfer from the upper (maternal) towards the lower (foetal) compartment was started by simultaneously replacing the buffer in both compartments. The buffer in the lower chamber was replaced with Na^+^‐free Hank's containing 300 μmol/L glutamine and 167 μmol/L unlabelled L‐leucine to obtain a physiological counter‐directed leucine gradient. Consecutively the buffer in the upper compartment was replaced with Hank's containing 30 μmol/L leucine (labelled with 3.7 nmol/L L‐[3,4,5‐^3^H(N)]‐leucine), 300 μmol/L glutamine and either vehicle (DMSO) or a constant 10 µmol/L‐dose of JPH203, JG336 or JX009, respectively. Inhibitors were applied in the upper compartment which is comparable to previous in vivo experiments.^40^ At defined time points between 5 minutes and 6 hours, 50 µL samples were taken from the maternal and foetal compartment. At the end of the experiment, all membranes were washed twice with DPBS and sampled to determine intracellular leucine levels. The medium and membrane samples including no cell controls were collected in 3 mL of scintillation cocktail (Zinsser Analytic, Frankfurt, Germany). Radioactivity was quantified using Tri‐carb 2100TR Liquid Scintillation Counter (PerkinElmer).

### 2.9 Statistical analysis

Anthropometric and clinical data are expressed as mean ± standard deviation (SD) for normal distribution or median with interquartile range for not normal distribution. Student t tests were performed to detect differences in mRNA levels between CTBs and STBs. MVM and BM membrane protein fractions were compared by using Mann‐Whitney test. A *P*‐value <0.05 was considered as statistically significant. Statistical comparisons were performed using GraphPad Prism software, La Jolla, USA.

## 3 RESULTS

### 3.1 LAT1, LAT2 and 4F2hc are asymmetrically expressed in the human placenta

Histological investigation of healthy placental tissue demonstrated strong expression of LAT1 at the apical membrane of STB (arrows in Figure 1B) which was confirmed by the strong signal found in the MVM fraction (Figure 1C). The asymmetric expression of LAT1 in syncytialized trophoblasts was further confirmed by immunoblot analysis in paired MVM/BM isolated from term control placentae. LAT1 was predominantly expressed at the MVM as reflected in a mean MVM to BM ratio of 6.9 (range 3.5‐14.9; Figure 1C). Western blot analysis of 12 MVM and BM pairs revealed a significantly higher expression of LAT1 in MVM compared to BM (*P* = 0.0015; Figure 1C). The results of these two independent experimental approaches suggest almost exclusive LAT1 expression at the apical membrane which is in direct contact with maternal blood. In contrast, LAT2 was found to be expressed in both MVM and BM (Figure S2), with an apparent predominance of the two LAT2 variants (30 and 50 kDa) in BM as reported before.^4^ Expression of 4F2hc, the heavy chain partner protein of LAT1 and LAT2, was higher in MVM as compared to BM (Figure S2).

### 3.2 Trophoblast differentiation induces up‐regulation of LAT1 and 4F2hc expression

**FIGURE 2:**
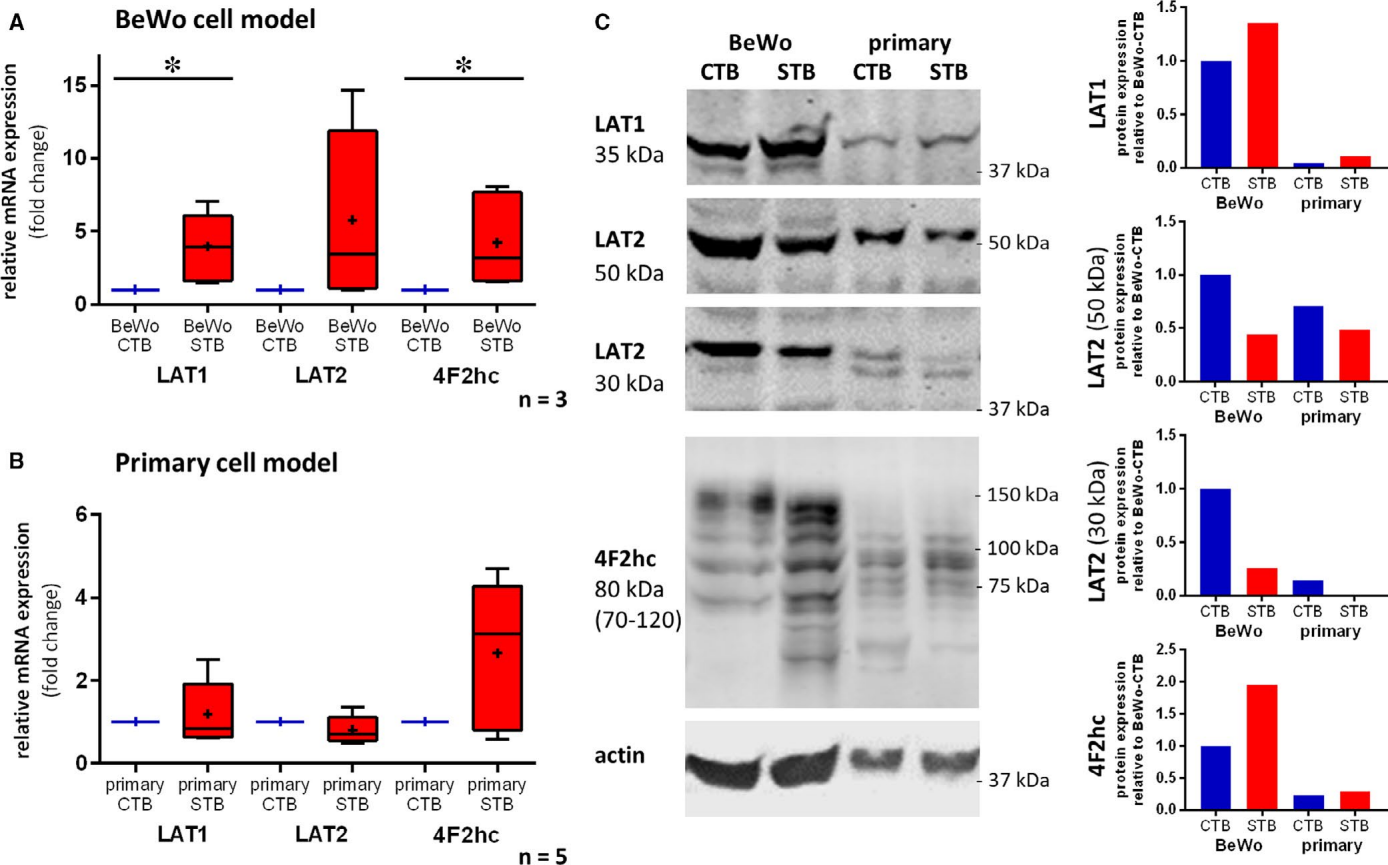
Expression patterns of the placental System L transporters in primary trophoblasts and BeWo cells depend on their differentiation stage. Comparison of the choriocarcinoma derived BeWo cell line (A) and primary trophoblasts (B) with respect to LAT1 (SLC7A5), LAT2 (SLV7A8) and 4F2hc (SLC3A2, CD98) mRNA levels at the undifferentiated cytotrophoblast (CTB) and differentiated syncytiotrophoblast (STB) stage. (A) LAT1 and 4F2hc were up‐regulated, while LAT2 expression in BeWo‐STB cells was not significantly increased relative to their BeWo‐CTB counterpart (paired t test of 3 independent experiments). BeWo‐CTB were sampled after 24 h of culturing and BeWo‐STB were analysed after stimulation with 100 μmol/L forskolin for 48 h. (B) LAT1, LAT2 and 4F2hc transcript levels of primary trophoblasts were not significantly increased in STB as compared to the CTB counterpart (paired t test). Primary trophoblasts were harvested from 5 individual trophoblast isolations (n = 5). They were lysed for mRNA isolation and quantitative RT‐qPCR after 12 h at the CTB stage and after 72 h of culturing at the STB stage. (A)/(B) Expression results were normalized to the mean of the reference genes YWHAZ, GAPDH and β‐actin. Transcript data are presented as fold‐change (2^−ΔΔCt^). Data are shown as mean (+), median (−) and Tukey whiskers (1.5‐times IQR), α = 0.05, *P < 0.05. (C) Representative immunoblot (left panel) and densitometric analysis of both cell types revealed increased LAT1 protein expression after cell differentiation (CTB < STB). Generally higher LAT1 protein expression levels were found in the BeWo cell line compared to primary cells (BeWo‐CTB/STB > pCTB/STB). The expression of the two LAT2 variants (50 kD; 30 kD) decreased after trophoblast differentiation in both cell types (primary trophoblasts and BeWo cells). 4F2hc was increased in both primary trophoblasts and BeWo‐STB as compared to the corresponding CTB stage. Densitometric analysis in the right panel was corrected for the β‐actin signal

To study the role of LAT1 at the placental barrier on the cellular level, LAT1 expression during trophoblast differentiation was analysed in both primary human trophoblast cells and in the BeWo cell line model on mRNA and protein level. Forskolin‐stimulated differentiation in BeWo cells resulted in a significant up‐regulation of LAT1 and 4F2hc, but no significant changes for LAT2 were observed (Figure 2A). Spontaneously occurring trophoblast differentiation in primary trophoblasts tended to increase LAT1 and 4F2hc mRNA levels, but due to the high variation between the individual placental cell isolations this effect did not reach statistical significance (Figure 2B). Interestingly, protein analysis by immunoblotting revealed a clear difference in the protein expression pattern between LAT1 and LAT2 depending on the differentiation state: while both 50 kDa and 30 kDa LAT2 variants were predominantly found in undifferentiated primary CTB and BeWo‐CTB, LAT1 protein levels were clearly increased in differentiated primary STB and BeWo‐STB (Figure 2C). Furthermore, increased 4F2hc expression was detected after differentiation in both primary and BeWo cells. 4F2hc protein expression was identified in the form of multiple bands ranging from 70 to 120 kDa under reducing conditions. The expression pattern of 4F2hc was found previously and is presumably due to formation of 4F2hc/4F2hc homodimers, 4F2hc/LAT2 heterodimers and 4F2hc monomers.^16^, ^41^, ^42^ BeWo cells exhibited higher expression levels, but comparable expression changes during differentiation as primary trophoblasts.

### 3.3 Leucine uptake increases with trophoblast differentiation

**FIGURE 3:**
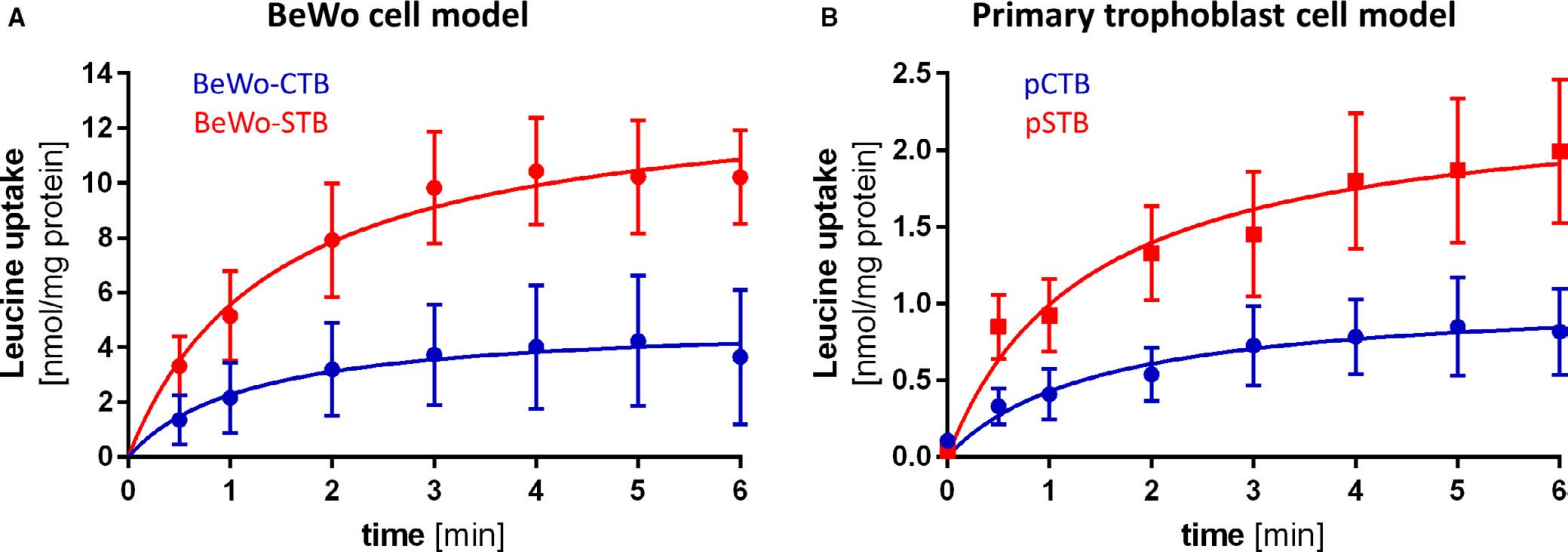
Similar leucine uptake kinetics in primary and BeWo trophoblast cells. A, In the BeWo cell model, Na^+^‐independent maximal uptake capacity (*V*_max_) for leucine was significantly increased in 3 independent experiments (n = 3). Leucine uptake increased from 4.96 nmol/mg protein in BeWo‐CTBs to 13.41 nmol/mg protein in BeWo‐STBs (2 way‐ANOVA, α = 0.05; *P* = 0.018). Leucine uptake for BeWo‐CTB was performed after 24 h of culturing and for BeWo‐STB after stimulation with 100 μmol/L forskolin for 48 h. B, Primary trophoblasts isolated from 3 individual control placentae (n = 3) significantly increased their *V*_max_ over 6 min from 1.046 nmol/mg protein in pCTB to 2.345 nmol/mg protein in the pSTB stage (2 way‐ANOVA, α = 0.05; *P* < 0.0001). The maximal leucine uptake capacity (*V*_max_) and half‐maximal uptake time (*K*_d_) was calculated using a saturation model curve fitting (*y* = *V*_max_*X/(*K*_d_ + *x*). The final concentration of leucine in all experiments was 167 nmol/mL including 1 µCi/mL ^3^[H]‐L‐leucine for detection. Error bars represent standard deviation (SD) of 3 individual experiments with 6 replicates each

We further investigated whether the expression changes caused by trophoblast differentiation resulted also in an increased leucine uptake efficiency in both primary (n = 3; Figure 3A) and BeWo cells (n = 3; Figure 3B). Indeed, both spontaneously differentiated primary trophoblasts (pCTB‐*V*_max_ = 1.05 nmol/mg protein vs pSTB‐*V*_max_ = 2.34 nmol/mg protein = 2.2‐fold) and forskolin‐stimulated BeWo cells (BeWo‐CTB‐*V*_max_ = 4.96 nmol/mg protein vs BeWo‐STB‐*V*_max_ = 13.41 nmol/mg protein = 2.7‐fold) reached more than 2‐times higher maximal uptake levels after 6 minutes compared to the undifferentiated stage. By comparing maximal leucine uptake levels between BeWo and primary cells, a 4.7‐fold greater uptake capacity for the BeWo‐CTB and 5.7‐fold difference for the BeWo‐STB stage was observed. However, the half‐maximal leucine uptake time, calculated as *K*_d_ from the time course curve fit, for primary cells (pCTB‐Kd = 1.45 nmol/min; pSTB‐hillslope = 1.36 nmol/min) and BeWo cells (BeWo‐CTB‐Kd = 1.19 nmol/min; BeWo‐STB‐Kd = 1.42 nmol/min) was similar between the differentiation stages. Thus, in accordance to expressional changes, also on functional level leucine uptake capacity was higher in BeWo cells compared to primary trophoblasts, but the kinetic changes due to cell differentiation were similar in both cell models (Figure 3).

### 3.4 Leucine uptake is modulated by System L‐specific small molecule inhibitors

**FIGURE 4:**
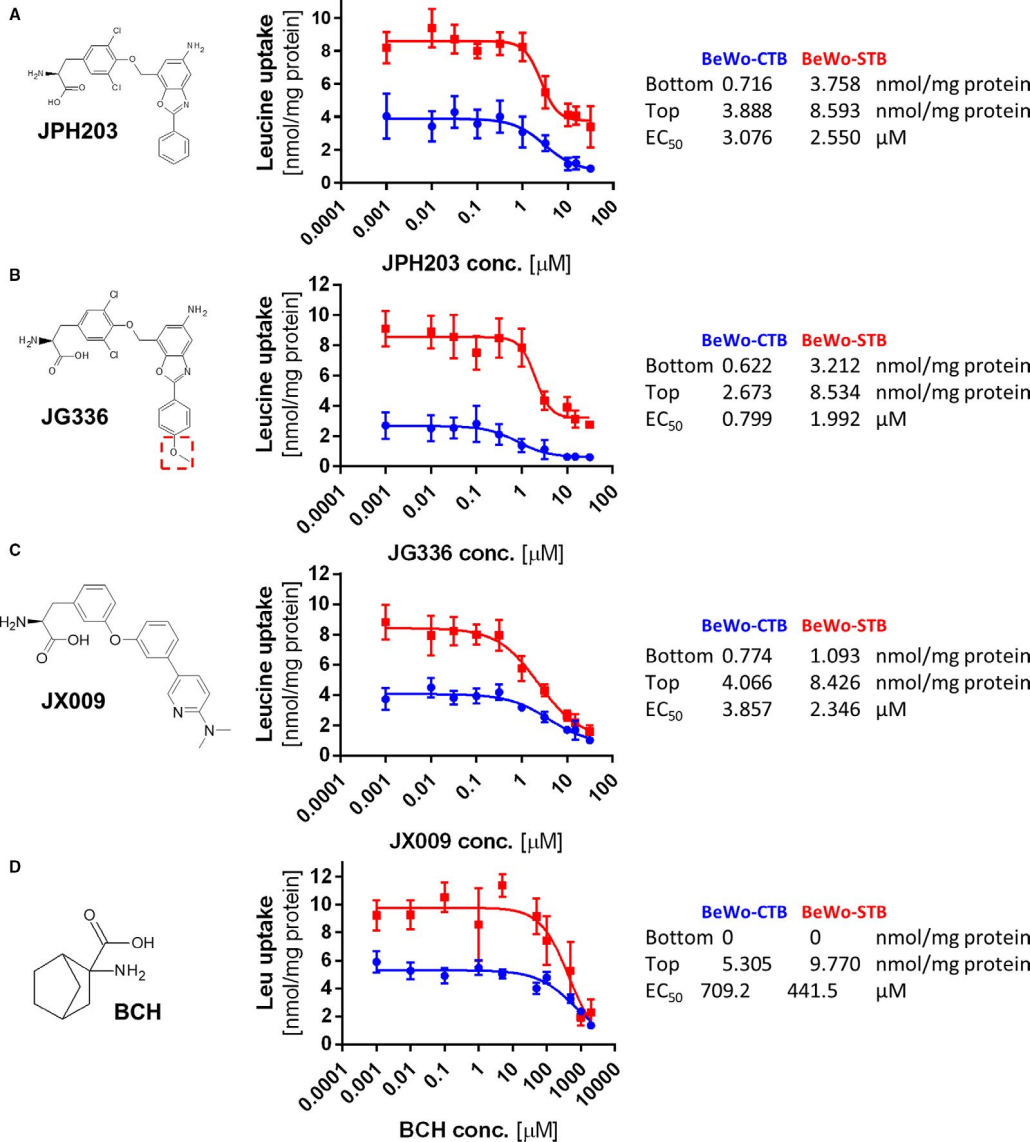
Inhibition of leucine uptake in BeWo cells using small molecule inhibitors with different specificities. Dose‐response experiments in BeWo‐cytotrophoblasts (BeWo‐CTB) and forskolin‐stimulated BeWo‐syncytiotrophoblasts (BeWo‐STB) for the established LAT1‐specific inhibitor JPH203 (A) and for the alternative compound JG336 which carries an additional methoxy group as marked in the dashed red box (B). The results showed that both compounds conveyed a significant inhibition (JPH203 = 56%, JG336 = 62%), but also suggest that approximately 40% of Na^+^‐independent leucine uptake is LAT1 independent. (C) Application of JX009, an inhibitor of both LAT1 and LAT2, resulted in maximal leucine uptake inhibition in BeWo‐CTB and BeWo‐STB. Compared to the dose‐response curve of the leucine analog 2‐amino‐2‐norbornane‐carboxylic acid (BCH, D), JX009 shows comparable efficiency but >180‐times higher potency. BCH is a widely accepted inhibitor blocking all System L mediated transport. BCH completely blocked leucine uptake in BeWo‐CTB with an EC_50_ of 709 µmol/L and BeWo‐STB with an EC_50_ of 442 µmol/L (D). (A‐C) The new inhibitors JG336 and JX009 as well as JPH203 reached maximal inhibition (first concentration without significant difference to the bottom value) at a concentration of 10 µmol/L, JG336 required 1 µmol/L for maximal inhibition in BeWo‐CTB. BCH reached maximal inhibition at 1 mmol/L in both differentiation stages. A‐D, All uptake assays were performed in two individual experimental setups (n = 2) for 3 min, under the same conditions in Na^+^‐free Hanks buffer with 167.2 µmol/L leucine (1 µCi/mL ^3^H‐L‐leucine). Dose‐response kinetics were calculated using the nonlinear four‐parameter model [Y = Bottom + (Top − Bottom)/(1 + 10^((LogEC50‐X)*HillSlope))] and ordinary fit with GraphPad Prism software. Best‐fit values of the including EC_50_ values are shown to the right of the dose‐response curves. Error bars represent standard deviation (SD) of 2 experiments with 6 replicates

Based on the high leucine uptake capacity and the high expression levels of functional LAT1, the BeWo cell model was selected to test different LAT1 or LAT1/LAT2‐specific small molecular inhibitors in dose‐response experiments. Two new alternative compounds, JX009 and JG336 (Figure 4B,C), were investigated and compared in two separate experiments to the already established potent LAT1‐specific inhibitor JPH203 (Figure 4A). In BeWo‐CTB all three inhibitors reached a maximal inhibition of less than 0.8 nmol/mg protein at a concentration of 10 µmol/L (corresponding to the bottom value of a four‐parameter nonlinear fit with variable slope; kinetic parameters are listed in the panels on the right). The leucine analog 2‐amino‐2‐norbornane‐carboxylic acid (BCH) maximally inhibited leucine uptake at a concentration of approx. 1 mmol/L regardless of the differentiation stage (Figure 4D). This observation further verifies the relevance of SLC7‐mediated leucine uptake in BeWo cells. Although there was profound leucine uptake inhibition in BeWo‐CTB, 10 µmol/L of JPH203 and JG336 showed a residual uptake of 3.8 (56.3% inhibition) and 3.2 nmol/mg protein (62.4% inhibition), respectively. JX009 (10 µmol/L) blocked leucine uptake regardless of the trophoblast differentiation stage. In BeWo‐STB leucine uptake was inhibited by 87% suggesting inhibition of most Na^+^‐independent SLC7 transporters. While the dose‐response experiments with the less specific inhibitor JX009 revealed an EC_50_ of 3.9 µmol/L for BeWo‐CTB and 2.3 µmol/L for BeWo‐STB, which is comparable with JPH203 (EC_50_ = 3.1 µmol/L for BeWo‐CTB and 2.6 µmol/L for BeWo‐STB), JG336 was identified as the most efficient inhibitor (EC_50_ = 0.8 µmol/L for BeWo‐CTB and 2.0 µmol/L for BeWo‐STB) in both differentiation stages of BeWo cells. Compared to the dose‐response curve of BCH, JX009 shows comparable efficiency but >180‐times higher potency (Figure 4C,D). The new inhibitors JG336 and JX009 as well as JPH203 reached maximal inhibition (first concentration without significant difference to the bottom value) at a concentration of 10 µmol/L, JG336 required 1 µmol/L for maximal inhibition in BeWo‐CTB. Of note, comparable inhibition patterns were also found in the colorectal adenocarcinoma cell line HT‐29 where lower substrate concentrations (30 µmol/L instead of 167 µmol/L leucine) lead to lower EC_50_ values (Figure S3). In summary, the dose‐response analysis of the three inhibitors suggested that JG336 inhibits LAT1‐specific placental leucine transport similar as JPH203, but exhibits an almost 4‐times higher efficiency. JX009, represents an inhibitor of System L transporters which in trophoblast is 180‐times more potent than BCH.

### 3.5 Transfer of leucine across the placental barrier is reduced by inhibitors of System L transporters

**FIGURE 5:**
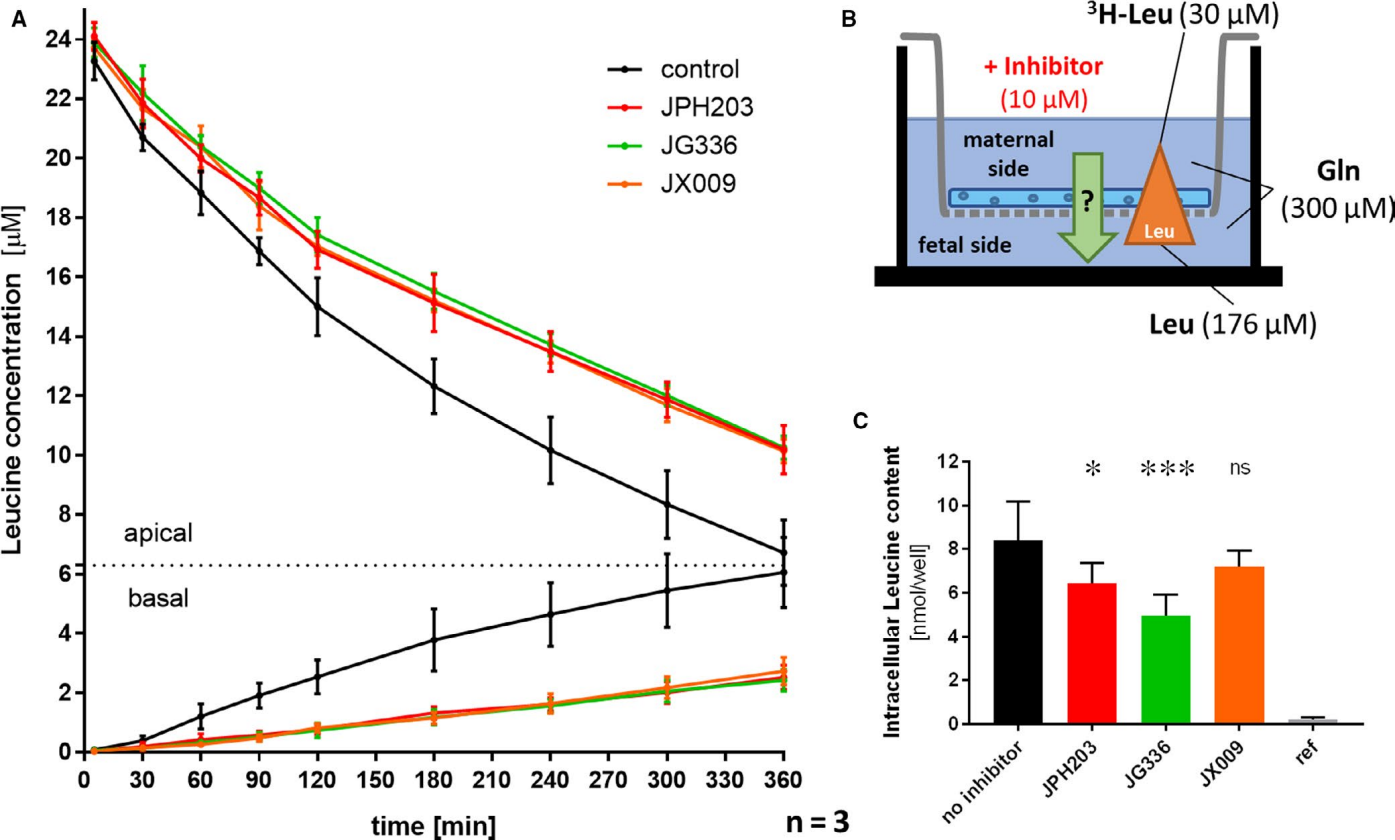
Relevance of LAT1 and System L inhibition in leucine transfer across the placental barrier in vitro. A, Leucine transfer across a tight BeWo monolayer was measured from the apical (ie upper compartment) towards the basal (ie lower compartment) side during 6 h using the Transwell^®^ system. Leucine transfer was significantly reduced by the System L inhibitor JX009 (orange) and the LAT1‐specific inhibitors JPH203 (red) and JG336 (green) compared to the DMSO‐control (black) already after 1 h. Equal concentrations (10 µmol/L) of each inhibitor and DMSO as vehicle control were applied in three experiments with two replicates per condition. B, Illustration of the experimental setup. All leucine transfer assays were performed against a counter‐directed gradient of 137 µmol/L (30 → 167 µmol/L) leucine, in the presence of 300 µmol/L glutamine (Gln) in both compartments. The leucine in the upper compartment was spiked with 1 µCi/mL ^3^H‐L‐leucine for transfer quantification. C, The intracellular leucine content was measured after washing the cell layers with DPBS at the end of the experiment (after 6 h). A significant reduction of intracellular leucine contents was detected for the LAT1‐specific inhibitors JPH203 (*P* = 0.020) and JG336 (*P* = 0.0001), but not for the System L inhibitor JX009‐ (*P* = 0.215). The detected intracellular leucine retention caused through LAT1 and LAT2 inhibition by JX009 suggests a System L‐dependent leucine efflux across the BM as rate‐limiting step. Statistical analyses were performed using a parametric one‐way ANOVA analysis (α = 0.05)

The small molecule inhibitors JPH203, JG336 and JX009 were also used to assess the relevance of LAT1 and LAT2 in leucine transport across the placental barrier using the Transwell^®^ system (Figure 5). Leucine transfer experiments were performed in the presence or absence of the individual inhibitors with a fixed concentration of 10 µmol/L applied at the upper compartment. This concentration was chosen as it showed in dose‐response experiments maximal inhibition for all three compounds (see above). Figure 5B shows the experimental setup and the applied leucine concentrations in the upper compartment (corresponding to the maternal side of the placental barrier) and the lower chamber (corresponding to the foetal side) of the Transwell^®^ system. Gradually diminishing radioactive signal ^3^[H]‐leucine on the maternal side and increasing radioactivity on the foetal side was considered to represent leucine transfer across the BeWo monolayer. Treatment with JPH203, JG336 and JX009 at the apical (maternal) side caused significant reduction of leucine transfer by 58%, 60% and 55%, respectively. All inhibitors significantly decreased leucine transfer from the apical towards the basal compartment from 60 minutes onwards. Measurement of intracellular ^3^[H]‐leucine content in the BeWo monolayer at the end of the transfer experiment (6 hours) indicated significantly decreased intracellular leucine concentrations of 24% for JPH203 and 41% for JG336, respectively (Figure 5C). In contrast, JX009 had no effect on the intracellular ^3^[H]‐leucine concentration within the tested time course. The higher retention of leucine in JX009 treated cells suggests inhibition of leucine secretion towards the lower foetal compartment.

## 4 DISCUSSION

The placenta plays a crucial role in the distribution of essential nutrients from the mother to the growing foetus. In this context, fine‐tuned regulatory mechanisms are needed to control the materno‐foetal transfer of essential AA to support the rapidly developing foetus during pregnancy.^43^ The current understanding of transplacental AA transport is based on the interplay between accumulative transporters such as members of the System A family and exchangers such as System L family members.^44^ System A family transporters like SNAT1, 2 and 4 are expressed at the MVM and accumulate small neutral AA against a concentration gradient in the STB in a Na^+^‐dependent manner.^43^ The AA accumulated by System A transporters can be used as substrates to exchange for essential large neutral AA such as leucine by the System L exchangers LAT1 and LAT2 across the MVM into the STB. The principles of AA uptake at the MVM are largely characterized, but the contribution of single transporters in AA transfer across the BM to the foetus is less clear.

In a first step, we investigated the expression and localization of LAT1 in the human placenta by immunohistochemistry of term placental tissue and by immunoblotting of MVM‐ and BM‐enriched membrane preparations. We found an exclusive apical expression of LAT1 at the MVM confirming previous reports.^8^, ^45^

So far most mechanistic studies are based on LAT1‐overexpressing cell models like human colon cancer‐derived HT‐29,^46^ mammary gland derived MCF‐7 cells,^47^ Pichia pastoris^42^ or reconstituted 4F2hc‐LAT1 proteoliposomes.^48^ Acquiring further detailed knowledge on the complex mechanisms regulating materno‐foetal AA transport at the placenta level could be beneficial to clarify the relevance of LAT1 and LAT2 in gestational diseases such as IUGR and LGA.^10^ To choose an appropriate physiological placental cell model, we compared the BeWo cell line with isolated primary human trophoblasts at different stages of differentiation by analysing LAT1/LAT2 mRNA and protein expression and leucine uptake. Spontaneous differentiation in primary trophoblasts and forskolin‐mediated differentiation in BeWo cells provoked expressional changes of LAT1, LAT2 and 4F2hc on mRNA and protein level (Figure 2). Our results demonstrated that the syncytialization process induced changes in the LAT1:LAT2 ratio as well as in 4F2hc expression in both trophoblast models (Figure 2) and resulted in an increased leucine uptake under Na^+^‐free conditions (Figure 3). These findings imply that differentiation induces a specialization process both in primary trophoblasts and BeWo cells, which results in an increased uptake, transport or transfer capacity as previously shown for the alanine‐serine‐cysteine transporters and for alpha‐aminoisobutyric acid transport.^49^, ^50^ This concept is in line with recent findings that differentiation processes such as syncytium formation resulted in an up‐regulation of MVM associated membrane proteins.^16^

Based on the validation of expression in the two trophoblast cell models (Figure 2) and comparable uptake behaviour (Figure 3), the BeWo cell line was chosen to test the effect of different SLC7‐specific inhibitors. However, to our knowledge LAT1 and LAT2 localization in MVM or BM of BeWo cells after forskolin‐induced differentiation has not been reported yet. Thus localization differences between primary trophoblasts and BeWo cells cannot be completely excluded and therefore the Transwell^®^ data using polarized BeWo cells should be interpreted with caution.

The low molecular weight inhibitors JPH203, JG336 and JX009 were assessed for their capacity to reduce leucine uptake into BeWo cells (Figure 4) and leucine transfer across the placental barrier (Figure 5). Due to the varying SLC7 specificity, different inhibition patterns were expected. JPH203 was previously reported as potent, LAT1‐specific inhibitor.^27^, ^28^, ^29^, ^30^, ^31^, ^46^ JX009 had been described in the patent literature^37^ to inhibit LAT1‐mediated transport with similar efficacy as JPH203, albeit with significantly lower specificity, that is, it is also a potent LAT2 inhibitor. As no other LAT2 inhibitors have been described in the literature, JX009 was considered the best available tool for the assessment of LAT2‐related transport of leucine. JG336 was prepared based on a limited structure activity relationships (SAR) study on JPH203 that investigated the effects of electron‐donating (such as the methoxy group in JG336, see dashed red box in Figure 4B), electron‐withdrawing and bulky substituents on the phenyl moiety at the 2‐position of the benzoxazole ring on transport inhibition. The additional methoxy group in JG336 resulted in 3.8‐times lower EC_50_ values in BeWo‐CTB (Figure 4B) indicating that the inhibition efficiency of JPH203 was indeed increased by this modification. Further results of this SAR study on JPH203 will be published separately.

Our results demonstrated that all three compounds are highly efficient LAT1 inhibitors, but only JX009 conveyed additionally LAT2 inhibition. Based on the complete leucine uptake inhibition of BeWo cells at the CTB stage, leucine uptake in undifferentiated trophoblasts seems to be highly dependent of LAT1 activity (Figure 4). Although we demonstrated in the expression studies (Figure 2) that the LAT1:LAT2 ratio in BeWo‐STB is higher compared to BeWo‐CTB, JPH203 reduced leucine uptake in BeWo‐STB by only 60%. This partial inhibition by JPH203 indicates that in BeWo‐STB leucine uptake is mediated by alternative Na^+^‐independent System L or System L‐like facilitators such as LAT2, LAT3 or LAT4. JX009 reduced leucine uptake by 87% regardless of the trophoblast differentiation stage with a low EC_50_ in the range of JPH203, suggesting that JX009 blocked most (if not all) Na^+^‐independent leucine uptake transporters. Thus, JX009 is the first characterized System L‐specific leucine uptake inhibitor in the low µM‐range (EC_50_ < 4 µmol/L; Figure 4) for use in placental cell models. To date, the most widely used SLC7‐specific inhibitor is the non‐metabolizable leucine analog BCH which has been also tested in this study (Figure 4D) and other investigations.^51^, ^52^ In comparison to JX009 this inhibitor is >100‐times less effective and blocks System L‐dependent AA transport with 709 µmol/L in BeWo‐CTB and 442 µmol/L in BeWo‐STB in the high µM‐range (Figure 4D). Nevertheless, further investigations of these small molecule compounds are needed to study viability, effects on AA metabolism and potential interactions with other transmembrane proteins such as System A transporters.

To assess the functional importance of SLC7 transporters in leucine transfer across the placental barrier, the effect of the LAT1‐specific inhibitors JPH203 and JG336 as well as the System L inhibitor JX009 was tested in the Transwell^®^ system with polarized BeWo cells grown in a tight monolayer (Figure 5). Treatment with JPH203 during 6 hours reduced leucine transfer across the placental barrier by 58%. Assuming 100% LAT1 inhibition after treatment with 10 µmol/L JPH203 (approximately 5‐times the EC_50_ of JPH203), these results suggest that more than half of the leucine transfer across the differentiated BeWo monolayer was LAT1‐dependent. The 3.8‐times more efficient inhibitor JG336 (60% reduction), and the less specific System L inhibitor JX009 (55% reduction) showed similar inhibition capacities as JPH203. These results demonstrated a predominantly LAT1‐dependent leucine transfer across the polarized BeWo monolayer which mimicks the physiological materno‐foetal barrier.^39^ In these transfer studies, we also measured the intracellular leucine content at the end of the experiment. In this context, the intracellular accumulation of leucine suggests that the efflux activity at the BM; that is, the transport towards the foetus, represents the rate‐limiting process in materno‐foetal transfer. In contrast, a reduced intracellular leucine content predicts leucine transport across the MVM into the BeWo monolayer as the rate‐limiting step.^7^ In our experiments only the two LAT1‐specific inhibitors JPH203 and JG336 caused a reduction of the intracellular leucine content (Figure 5C), indicating that LAT1‐mediated leucine transfer occurs at the apical MVM. In contrast, the unchanged intracellular leucine content after JX009 treatment suggests System L transporter‐dependent leucine efflux across the BM. This is in agreement with the hypothesis that LAT1 is the major leucine transporter at the MVM, while other placental Na^+^‐independent System L or System L‐like facilitated AA transporters such as LAT2, LAT3 or LAT4 mediate leucine efflux across the BM (Figure 1) as previously proposed.^7^, ^43^, ^53^, ^54^ LAT1 and LAT2 localization in BeWo cell before and after differentiation has not been demonstrated yet; hence the interpretation of our results is based on the assumption that System L‐transporter expression in differentiated BeWo is similar to primary trophoblasts and placental tissue. Since the three tested inhibitors were applied at the maternal compartment, there is a certain membrane and cell layer permeability required to allow interactions with the transporters located at the BM. Permeability across the trophoblast barrier has not been investigated, but has been shown in Caco‐2 cells for JPH203.^40^ Recently Lewis et al demonstrated in a mathematical modelling approach of placental AA efflux based on placental perfusion data, that LAT2 allows the transport of a substrate across the BM without transport of another molecule in the other direction as normally expected from a classical exchanger.^55^

The selective expression and overexpression in various cancer types with poor survival expectancy^20^, ^56^, ^57^, ^58^, ^59^, ^60^ made LAT1 an interesting pharmaceutical target.^27^ This study demonstrates that the choriocarcinoma cell line BeWo could serve as useful model system to test putative LAT1 inhibitors and characterize their effect at an active AA transporting and physiologically relevant cell barrier. It was shown that high and asymmetric expression of SLC7 family members makes the placental leucine uptake sensitive to LAT1‐ and LAT2 inhibition.^8^, ^41^ Furthermore, the application of compounds like the LAT1‐specific inhibitor JPH203 or the less specific SLC7‐transporter inhibitor JX009, allows for distinguishing the contribution of single leucine transporters across the placental barrier. The short‐term treatment with small molecule inhibitors also reduces the risk of affecting trophoblast fusion and differentiation which could occur after long‐term gene silencing or constitutive knockout.^16^ If further efforts are made to identify new lead structures or to further develop existing inhibitors targeting System L‐like transporters such as the AA facilitators LAT3 and LAT4, these new compounds could be valuable tools for assessing the relevance of single transporter in materno‐foetal AA transfer.

In summary, the combined application of the LAT1‐specific inhibitor JPH203 and the System L inhibitor JX009 in Transwell^®^ studies helped to identify LAT1 as major leucine transporter playing an important role in the materno‐foetal supply of essential AA at the MVM. In this context, the present study demonstrates the usefulness of applying small‐compound inhibitors bearing different specificities. A better understanding of the SLC7 transporter‐mediated supply of AA and its impact on mTORC1‐mediated placental function in pregnancy diseases including IUGR and gestational diabetes mellitus could be a first step towards the optimization of foetal growth and its effect on foetal programming in the future.^61^

## CONFLICT OF INTEREST

The authors declare no conflict of interest.

## AUTHOR CONTRIBUTION

**Jonas Zaugg:** Conceptualization (equal); data curation (lead); formal analysis (lead); investigation (lead); methodology (lead); validation (lead); writing – original draft (lead); writing – review and editing (equal). **Xiao Huang:** Conceptualization (equal); data curation (equal); investigation (equal); writing – review and editing (equal). **Fabian Ziegler:** Data curation (equal); formal analysis (equal); validation (equal). **Matthias Rubin:** Data curation (equal); formal analysis (supporting). **Julien Graff:** Data curation (equal); formal analysis (supporting). **Jennifer Müller:** Data curation (equal); formal analysis (supporting). **Ruedi Moser** – **Hässig:** Conceptualization (equal); methodology (equal); writing – review and editing (equal). **Theresa Powell:** Methodology (equal); writing – review and editing (equal). **Jürg Gertsch:** Supervision (equal); writing – review and editing (equal). **Karl‐Heinz Altmann:** Resources (equal); validation (equal); writing – review and editing (equal). **Christiane Albrecht:** Conceptualization (lead); formal analysis (supporting); funding acquisition (lead); investigation (supporting); project administration (lead); resources (equal); supervision (lead); writing – original draft (equal); writing – review and editing (lead).

## Supporting information

Supplementary Material

## Data Availability

The data that support the findings of this study are available from the corresponding author upon reasonable request.
