# Supplementary material for: Small molecule inhibitors provide insights into the relevance of LAT1 and LAT2 in materno‐foetal amino acid transport
Source: J Cell Mol Med. 2020 Oct 1;24(21):12681–93. doi: 10.1111/jcmm.15840 (PMC7687008; doi:10.1111/jcmm.15840)
Supplement: Supplementary file 1 — Supplementary Material [file JCMM-24-12681-s001.docx]

Small molecule inhibitors provide insights into the relevance of LAT1 and LAT2 in materno-fetal amino acid transport

by Zaugg et al., submitted *to Journal of Cellular and Molecular Medicine*

**Supplemental information**

## Placental membrane protein isolation

In brief, approximately 100g villous tissue from central and lateral areas of the placenta was washed 3-times with NaCl 0.9% to remove blood. All following steps were performed at 4°C or on ice. After the final wash, the tissue was homogenized in Buffer H (250mM sucrose, 10mM Hepes, adjusted pH to 6.95, supplemented with protease inhibitor cocktail) for 2min with a Polytron homogenizer (Kinematica AG). The crude homogenate was centrifuged with 1000rcf to remove cellular debris. Two differential centrifugation steps at 10’000rcf for 15min at 4°C (Optima L-90K ultracentrifuge with TFT70.38 rotor) were performed. The supernatants of both centrifugations were combined and filtered through 4 layers of gauze on ice and then centrifuged at 125’000rcf for 30min at 4°C (TFT 70.38 rotor). The pellets containing all cellular membranes were homogenized with a Teflon homogenizer and diluted with Buffer H, followed by Mg^2+^ precipitation applying a final Mg^2+^ concentration of 12mM by adding 240mM MgCl_2_ and slowly stirring for 20min. The centrifugation with 2500rcf for 10min separates MVM in the supernatant and all other membranes in the pellet. To isolate the BM-fraction from the pellet by ultracentrifugation on a 2 step gradient, it was first washed, then resuspended with Buffer E (0.1% bovine serum albumin in 1mM Titriplex® III, Merck, Darmstadt, Germany) and homogenized using a Teflon homogenizer. A two-step sucrose gradient was prepared in a thin-walled ultra-centrifuge Beckman SW28 tube (Beckman Coulter, Brea, CA, USA) by applying a bottom layer with 1.192g/cm^3^ density, a middle layer with 1.162g/cm^3^ density and on top the homogenized membrane-fraction. This gradient was centrifuged at 100’000rcf for 60min using a Beckman SW28 swing-out rotor (Beckman Coulter, Brea, CA, USA). The BM deposit between the bottom and the middle layer was removed. Finally, the BM-fraction and the MVM pellets were resuspended in buffer E and homogenized with a Teflon homogenizer. MVM and BM were centrifuged at 125’000rcf for 30min for a final clean-up. The pellets were resuspended in buffer H, snap frozen in liquid nitrogen and stored at -80°C.

## Cryo-sectioning and immunohistochemistry

Tissues from control-placentae were embedded in Tissue-Tek^®^ optimum cutting temperature (O.C.T.) medium, cut at 5µm thickness and mounted on Superfrost plus slides (Menzel, Germany). The frozen tissue blocks were stored at -80°C upon sectioning. The tissue sections were fixed in pre-cooled acetone (-20°C) and washed twice in 10mM phosphate buffered saline (PBS, pH 7.4). Fixed sections were incubated in H_2_O_2_-Block (Dako S2023) at RT for 10min to block endogenous peroxidase activity and again washed twice in PBS. The primary antibodies were diluted in PBS with 0.5% bovine serum albumin. Diluted primary antibodies were added to cover the tissue on the slides and incubated in a humidified chamber 1.5h at 4°C. For the visualization of the antigens, UltraVision LP Detection System (Thermo Scientific™ TL-015-HAS) was used according to the manufacturer’s instructions. Slides were counterstained with hematoxylin (Sigma) and mounted with Aquatex^®^ (EMD Millipore 1.08562). Negative controls were stained without prior incubation with the primary antibody. Immunohistochemical images were prepared with a DM6000 B microscope (Leica Microsystems, Germany).

## LAT1 expression by immunoblotting

Primary trophoblasts and BeWo cells were lysed by adding hypotonic lysis buffer (10mM Tris-HCl, Sigma T5941; 10mM NaCl, Sigma 71376; 1.5mM MgCl_2_, Millipore 1.05833; 1% Triton X-100, Sigma T8787; 1 bottle/100mL protease inhibitor cocktail, Sigma P2714; adjusted pH to 7.4). The samples were vortexed in hypotonic lysis buffer every 5min during 30min on ice for thorough lysis of the cells. Subsequently, the cell lysates were centrifuged at 1000 x g for 10min at 4°C to remove cellular debris and stored upon analysis at -20°C. Protein content was measured using the Pierce™ BCA Protein Assay Kit.

50µg cell lysates were loaded on 10% acrylamide gels and separated by SDS–PAGE using the Biorad minigel system. Sample preparation was performed for SDS-PAGE using 5x Laemmli sample buffer (400mM Tris-HCl, Sigma T594; 50% (w/v) Glycerol, Sigma G6279; 10% (w/v) SDS, Sigma L6026; 500mM DTT, Sigma D9779; 0.05% (w/v) Bromophenol Blue, Sigma B8026), but we are not boiling the samples. The immobilized bands were then semi-dry transferred to nitrocellulose membranes (GE Healthcare). Blots were blocked with 5% non-fat milk in Tris Buffered Saline with 0.1% Tween-20 (TBST). The primary polyclonal rabbit anti-LAT1 antibody (KE026/TG170215 Transgenic Inc.) and for the reference signal (loading control) the mouse anti-beta-actin antibody (Sigma A2228) were incubated overnight at 4°C, followed by 4 times washing with TBST, and incubation with DyLight 680 or 800 fluorescence conjugated secondary antibodies (Thermo Scientific™). The immunoreactive bands were quantiﬁed with the OdysseyW Sa Infrared Imaging System (LI-COR) to obtain relative densitometry values without signal saturation.

**Calculation of the apparent permeability coefficient in the BeWo Transwell^®^ experiments**

The apparent permeability coefficient (Papp) was calculated according to following equation:

$$Papp\left[ \frac{cm}{s} \right]=\frac{dQ}{dt} \div(A \times C_{0})$$

dQ/dt: Rate of substrate transport from donor to receiver chamber [μmol/s]

A: Surface area of the filter membrane [cm^2^]

C_0_: Initial concentration of the substrate in the donor chamber [μmol/mL]

## Analytical data of inhibitors

### JPH203:

**^1^H-NMR** (400 MHz, DMSO-d_6_): δ (ppm) = 8.13 – 8.07 (m, 2H), 7.62 – 7.57 (m, 3H), 7.42 (s, 2H), 6.90 (d, J = 2.1 Hz, 1H), 6.85 (d, J = 2.0 Hz, 1H), 5.19 (s, 2H), 3.46 – 3.43 (m, 1H), 3.08 (dd, J = 14.3, 4.6 Hz, 1H), 2.87 (dd, J = 14.3, 7.8 Hz, 1H).

**^13^C-NMR** (101 MHz, DMSO-d_6_): δ (ppm) = 169.0, 161.9, 148.7, 146.7, 142.7, 141.3, 136.4, 131.6, 130.3, 129.3, 128.3, 126.9, 119.1, 114.0, 103.0, 69.6, 55.0, 35.7.

### JG336:

**^1^H-NMR** (400 MHz, DMSO-*d_6_*): δ (ppm) = 8.03 (d, *J* = 8.8 Hz, 2H), 7.45 (s, 2H), 7.14 (d, *J* = 8.8 Hz, 2H), 6.86 (d, *J* = 1.8 Hz, 1H), 6.80 (s, 1H), 5.16 (s, 2H), 3.85 (s, 3H), 3.17 – 2.84 (m, 2H).

**^13^C-NMR** (101 MHz, DMSO-*d_6_*): δ (ppm) = 161.9, 148.7, 146.5, 142.9, 141.2, 130.3, 128.7, 128.2, 119.2, 118.8, 114.8, 113.3, 102.9, 69.7, 55.5, 54.9, 31.3, 29.0.

**HRMS** (ESI) calculated for C_24_H_22_Cl_2_N_3_O_5_ [M+H^+^] 502.0931, found 502.0929.

### JX009:

**^1^H-NMR** (400 MHz, CD_3_OD): δ (ppm) = 8.30 (dd, *J* = 9.6, 2.3 Hz, 1H), 8.14 (dd, *J* = 2.3, 0.7 Hz, 1H), 7.50 (t, *J* = 7.9 Hz, 1H), 7.42 (ddd, *J* = 7.8, 1.8, 1.0 Hz, 1H), 7.40 (t, *J* = 7.9 Hz, 2H), 7.34 (dd, *J* = 9.6, 0.7 Hz, 1H), 7.31 (t, *J* = 2.1 Hz, 1H), 7.12 (dt, *J* = 7.8, 1.1 Hz, 1H), 7.08 – 7.01 (m, 2H), 6.99 (ddd, *J* = 8.1, 2.4, 1.0 Hz, 1H), 4.28 (dd, *J* = 7.2, 5.9 Hz, 1H), 3.35 (s, 6H), 3.31 (dd, *J* = 14.0, 6.0 Hz, 1H), 3.20 (dd, *J* = 14.4, 7.1 Hz, 1H).

**^13^C-NMR** (101 MHz, CD_3_OD): δ (ppm) =171.20, 159.43, 158.78, 153.10, 143.35, 137.97, 137.77, 134.32, 132.04, 131.72, 126.05, 125.89, 122.33, 121.38, 121.13, 119.74, 119.38, 117.62, 113.81, 55.10, 39.92, 37.13.

**HRMS** (ESI) calculated for C_22_H_24_N_3_O_3_ [M+H]^+^ 378.1812, found, 378.1813.

**Supplemental Table 1:** List of primer sequences used to quantify LAT1 expression in trophoblasts relative to reference genes by RT-qPCR.

| Gene | Sequence [5'-3'] | length | fragment length [bp] |
| --- | --- | --- | --- |
| LAT1 | CAGGGCATCTTCTCCACGAC | 20 | 137 |
|  | TGGGTTCGAGGAGGTGATCTA | 21 |  |
| LAT2 | AGGCCCTCCTCTGTGGCTGG | 22 | 106 |
|  | GTGCAGCAGGAGTGGCTGGG | 20 |  |
| 4F2hc | AGCTGGAGTTTGTCTCAGGC | 20 | 127 |
|  | GGCCAATCTCATCCCCGTAG | 20 |  |
| YWHAZ | CCGTTACTTGGCTGAGGTTG | 20 | 143 |
|  | AGTTAAGGGCCAGACCCAGT | 20 |  |
| Beta actin | AACTCCATCATGAAGTGTGACG | 22 | 234 |
|  | GATCCACATCTGCTGGAAGG | 20 |  |
| GAPDH | ACCACAGTCCATGCCATCAC | 20 | 452 |
|  | TCCACCACCCTGTTGCTGTA | 20 |  |

# Figure legends Supplemental Figures

## Suppl. Figure 1: Characterization of MVM/BM enrichment by determining alkaline phosphatase activity and Ferroportin expression

Alkaline phosphatase is a marker enzyme for the syncytiotrophoblast (STB) apical membrane/microvillous membrane (MVM)^a^, while Ferroportin 1 (FPN1) as iron exporter is expressed in the basal membrane (BM)^b^. Alkaline phosphatase activity in MVM and BM samples from membrane isolations were compared to their corresponding homogenate fraction before MVM/BM enrichment using a parametric paired t-test. Alkaline phosphatase activity relative to homogenate as depicted in **A** was 7.0-times higher in MVM (9.6-times enrichment) compared to BM (1.6-times enrichment). **B** left panel shows p-nitrophenyl phosphate (pNPP) hydrolysis (colorless) to p-nitro-phenol (yellow, λ_abs._=405nm) by alkaline phosphatase activity of a representative experiment during 2min. FPN1 expression was analyzed by immunoblotting (**C**). BM-marker FPN1 was increased in BM compared to MVM fractions. Characterization of MVM/BM enrichment was performed for 6 out of 11 isolations shown in Fig.1.

^a^ Illsley NP, Wang ZQ, Gray A, Sellers MC, Jacobs MM. Simultaneous preparation of paired, syncytial, microvillous and basal membranes from human placenta. BBA - Biomembr. 1990;1029(2):218–26.

^b^ Bastin J, Drakesmith H, Rees M, Sargent I, Townsend A. Localisation of proteins of iron metabolism in the human placenta and liver. Br J Haematol. 2006;134(5):532–43.

## Suppl. Figure 2: Asymmetric expression of leucine transporters in the human placenta

The expression of LAT1 (SLC7A5), LAT2 (SLC7A8) and 4F2hc (SLC3A2) was analyzed in total membrane isolation (TMI), microvillous membrane (MVM) and basal membrane (BM) fractions in two independent experiments (n=2) by immunoblotting. In the left panels images of a representative immunoblot with densitometric quantification are shown. On the right panels the densitometric analyses and consecutive statistics for both experiments are shown. LAT1 was almost exclusively expressed in MVM, while LAT2 was found to be expressed in both MVM and BM. 4F2hs, the heavy chain partner protein of LAT1 and LAT2, was higher expressed in MVM as compared to BM. Beta-actin was used as reference gene.

## Suppl. Figure 3: Leucine uptake inhibition and substrate-dependence of EC_50_ values in HT-29 cells using SLC7 inhibitors.

Dose-response experiments for leucine uptake inhibition in the colorectal adenocarcinoma cell line HT-29 were performed in Na^+^-free Hanks buffer with 167µM leucine and 1µCi/mL ^3^H-L-leucine (left panels A, B and C) and in Na^+^-free Hanks buffer with 30µM leucine and 1 µCi/mL ^3^H-L-leucine (right panels A, B and C). HT-29 cells and a substrate concentration of 30µM L-leucine are usually used to compare EC_50_ values in structure–activity relationship studies. Dose-response experiments demonstrated complete leucine uptake inhibition with JPH203 (A left panel, EC_50_ 2.17µM) and the two novel SLC7-targeting inhibitors JG336 (B left panel, EC_50_ 0.73µM) and JX009 (C left panel, EC_50_ 3.76µM) under physiological 167µM leucine concentrations. JG336 was the most efficient inhibitor being 2.6-times more efficient than the LAT1-specific inhibitor JPH203. All tested inhibitors show markedly lower EC_50_ values at the lower substrate concentration of 30µM, i.e. for JPH203 (A right panel) EC_50_=0.15µM, JG336 (B right panel), EC_50_=0.21µM and JX009 (C right panel), EC_50_=0.60µM. The leucine uptake experiments were performed during 3min in Na^+^-free Hanks buffer with 167µM leucine (1µCi/mL ^3^H-L-leucine) and are shown as representative experiments of two independent experiments (n=2). Dose-response kinetics were calculated using same statistical tools as explained in Fig.4. EC_50_ values are shown to the right of the dose-response curve. Error bars represent standard deviation (SD) of 2 experiments with 6 replicates.

# Supplemental Figures


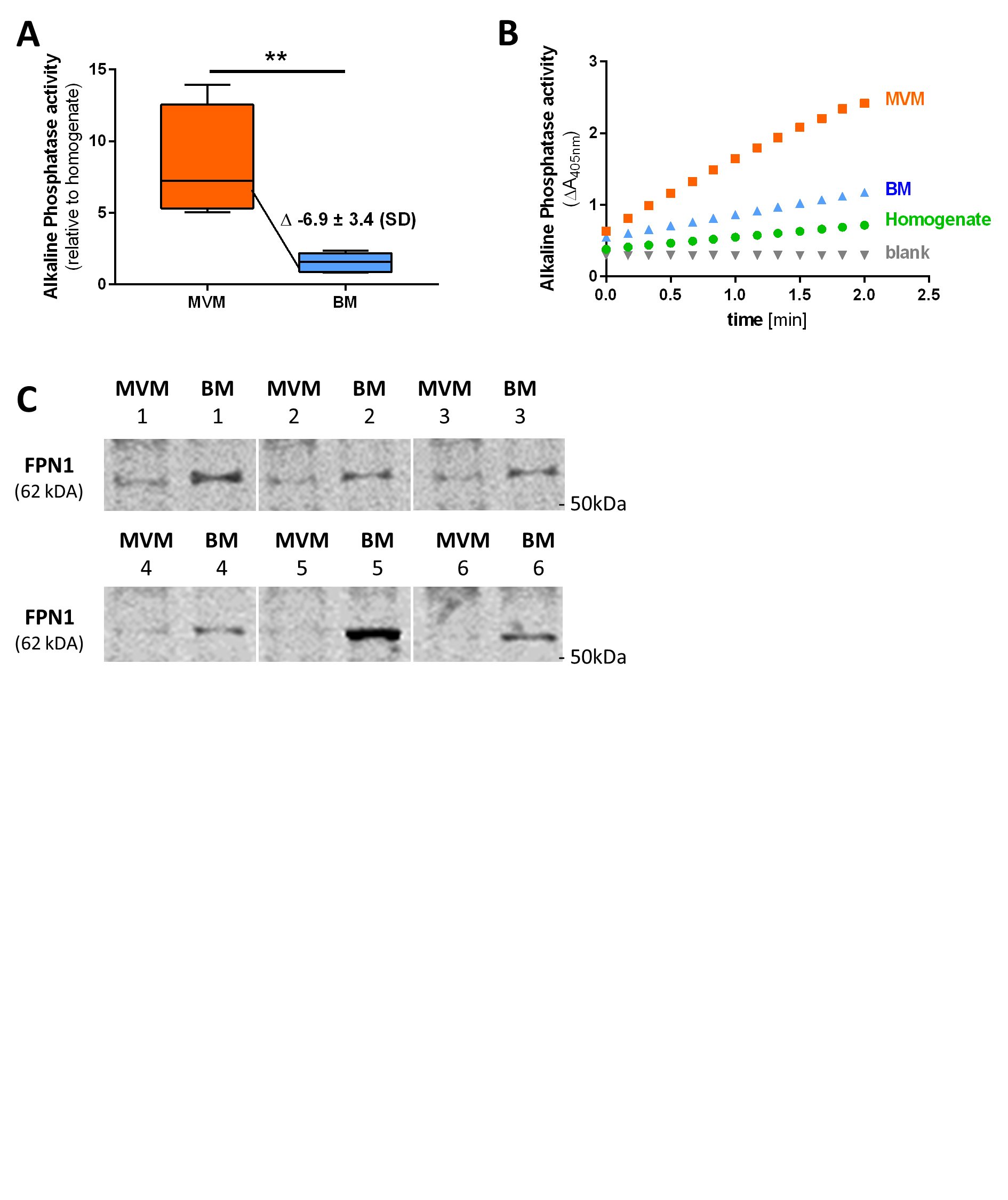


## Supplemental Figure 1


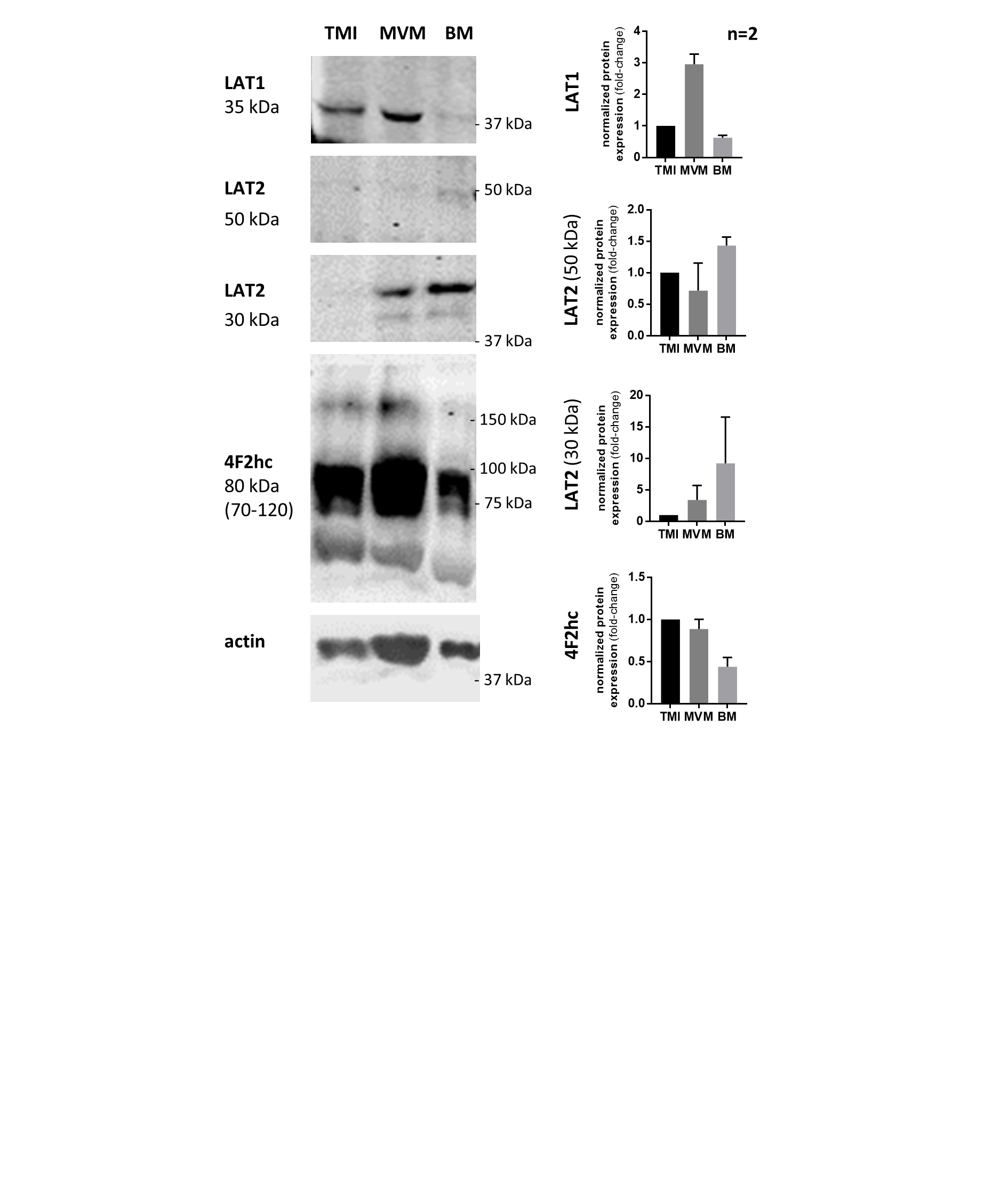


## Supplemental Figure 2


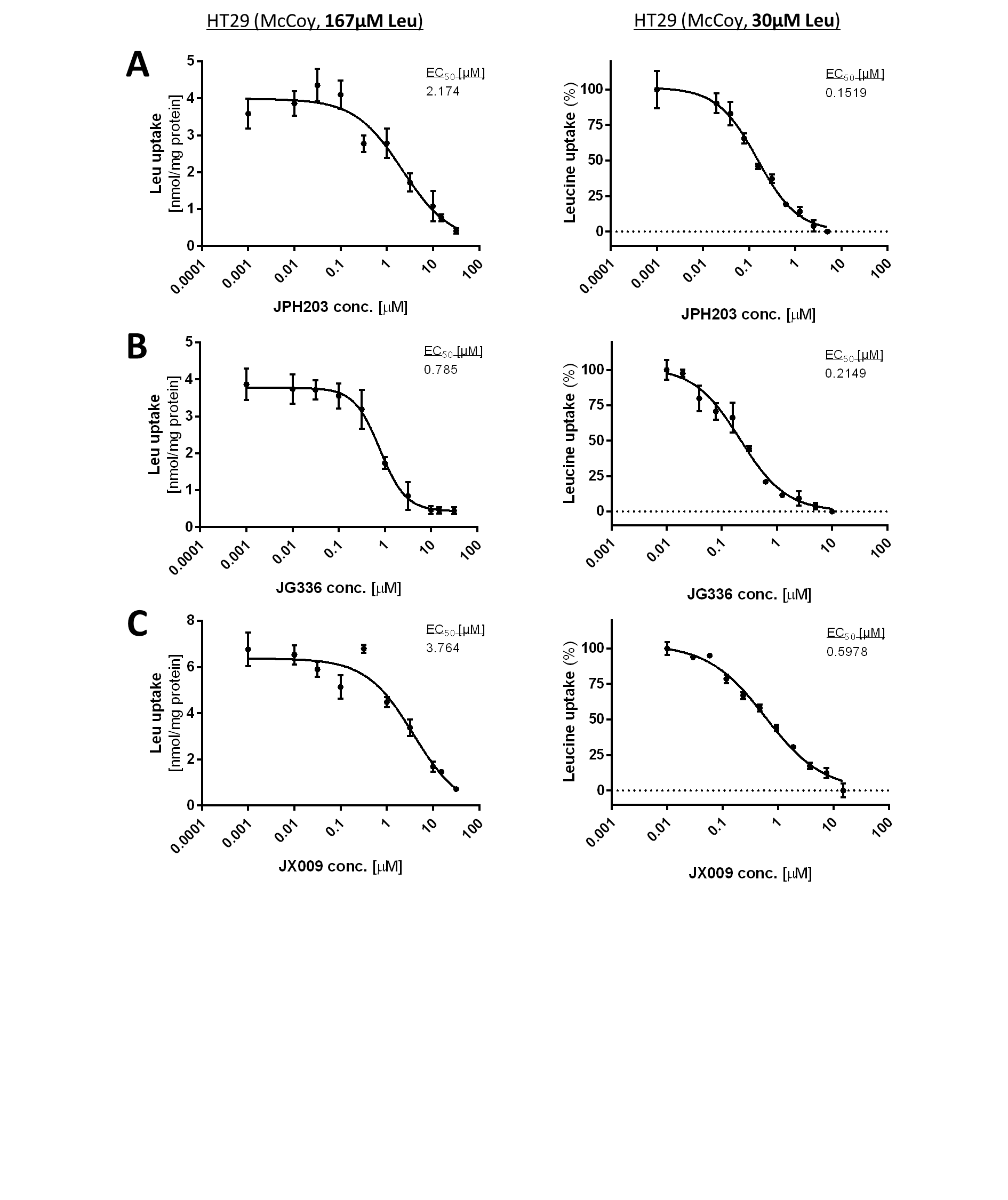


## Supplemental Figure 3
